# The Effect of Governmental Health Measures on Public Behaviour During the COVID-19 Pandemic Outbreak

**DOI:** 10.34172/ijhpm.2021.131

**Published:** 2021-09-11

**Authors:** Guoyan Wang, Li Li, Lingfei Wang, Zhi Xu

**Affiliations:** ^1^School of Communication, Soochow University, Suzhou, China.; ^2^Health Inspection Institute, Health Commission of Suzhou, Suzhou, China.; ^3^Jiangsu Key Laboratory of Culture and Tourism for Digital Twin Perception Technology in Museums, Suzhou, China.

**Keywords:** COVID-19, Government Measure, Epidemic Management, Public Behaviour, Mobility Restriction, Large-Scale Data

## Abstract

**Background:** The coronavirus disease 2019 (COVID-19) pandemic resulted in radical changes in many aspects of life. To deal with this, each country has implemented continuous health measures from the beginning of the outbreak. Discovering how governmental actions impacted public behaviour during the outbreak stage is the purpose of this study.

**Methods:** This study uses a hybrid large-scale data visualisation method to analyse public behaviour (epidemic concerns, self-protection, and mobility trends), using the data provided by multiple authorities. Meanwhile, a content analysis method is used to qualitatively code the health measures of three countries with severe early epidemic outbreaks from different continents, namely China, Italy, and the United States. Eight dimensions are coded to rate the mobility restrictions implemented in the above countries.

**Results:** (1) Governmental measures did not immediately persuade the public to change their behaviours during the COVID-19 epidemic. Instead, the public behaviour proceeded in a three-phase rule, which is typically witnessed in an epidemic outbreak, namely the wait-and-see phase, the surge phase and the slow-release phase. (2) The strictness of the mobility restrictions of the three countries can be ranked as follows: Hubei Province in China (with an average score of 8.5 out of 10), Lombardy in Italy (7.125), and New York State in the United States (5.375). Strict mobility restrictions are more likely to cause a surge of population outflow from the epidemic area in the short term, whereas the effect of mobility restrictions is positively related to the stringency of policies in the long term.

**Conclusion:** The public showed generally lawful behaviour during regional epidemic outbreaks and blockades. Meanwhile public behaviour was deeply affected by the actions of local governments, rather than the global pandemic situation. The contextual differences between the various countries are important factors that influence the effects of the different governments’ health measures.

## Background

**Key Messages**

** Implications for policy makers**

- This study provides a valuable reference for policy-makers with regard to the timing, content, and form of measures, and could be referred to during epidemic management situations in the future.
- There is typically a three-phase rule of public behaviour in an epidemic outbreak, namely the wait-and-see phase, the surge phase and the slow-release phase.
- Strict mobility restrictions are more likely to cause a surge of population outflow from the epidemic area in the short term.
- The effect of mobility restrictions is positively related to the stringency of restriction policies in the long term.
- Out of all government measures, a regional blockade sends the strongest epidemic signal, which in turn leads to the most drastic changes in local public behaviour in all indices.
- Public behaviour is deeply affected by the actions of local governments, rather than the global pandemic situation.

** Implications for the public**

This study provides an opportunity to gain insight into policies or health measures and public behaviours. Based on the study of public behaviour, individuals can adapt their own behaviour to better protect themselves against a public crisis. First, this study finds that strict mobility restrictions are more likely to cause a surge of population outflow from the epidemic area in the short term. On the premise of monitoring health, understanding the above can help individuals decide whether or not to go out at this time, as well as when it would be better to go out. Second, the behaviour of the public is largely affected by local governments. Therefore, if the outbreak of a local epidemic occurs later in one country than in other countries, the later government’s actions will lag behind. For better self-protection, the public should try to pay close attention to the dynamics of the countries suffering early epidemic outbreaks, in order to guide their own preventive action.

The pandemic caused by the virus severe acute respiratory syndrome coronavirus 2 (SARS-CoV-2) has spread globally. As of August 16, 2021, there have been more than 200 million infections and 4.3 million deaths,^1^ and the epidemic is still not over.

 Governments and professional health organizations around the world have responded to the epidemic by promulgating and carrying out a range of management measures. Cheng et al^2^ documented over 13 000 such policy announcements across more than 195 countries, including policy types such as quarantine (or lockdown), social distancing, public awareness measures, etc. The implementation of different measures in different countries has also been compared.^3,4^ In terms of the impact of government measures, most articles were based on mathematical models (eg, SEIR), and used the COVID-19 cumulative incidence^5^ or effective reproductive number (R)^6^ as an assessment term. For example, Gatto et al^7^ found that control of human movement would reduce the spread of the outbreak by 45%, while the prediction model of Yang et al^8^ presented a potential scenario that a 5-day delay in implementing policies such as mass quarantine, travel restrictions, suspected case detection, and tracing would triple the size of the epidemic in China. Furthermore, Haug et al^9^ ranked the effectiveness of worldwide government interventions.

 Notably, a study of the Ebola outbreak found that behavioural changes were the core driver in the epidemic’s decline.^10^ With regard to the coronavirus disease 2019 (COVID-19)pandemic, Roma et al^11^ based their studies on the observation that Italian residents practiced incongruous behaviours with regard to the government’s measures. These studies all stressed the critical importance of public compliance to helping control the epidemic. Finset et al^12^ argued that, although people are already aware of policy recommendations (such as frequent hand washing and social distancing), it is not easy to go from knowing these things and doing them, due to the gap between intention and behaviour. With regard to this discrepancy, former studies showed that changes in intention produce comparatively smaller changes in behaviour.^13,14^ Generally speaking, public awareness of policies does not necessarily lead to behavioural changes. As to the impact of government policies and measures on people’s behaviour, a number of scholars have conducted studies through survey methods (such as questionnaires), and by investigating people’s actions, including wearing masks^15^ and maintaining social distance.^16^ More specifically, Zhong et al^17^ collected 6910 online questionnaires in China. The study found that the vast majority of participants had not visited any crowded place (96.4%), and an even larger percentage wore masks when going out (98.0%). However, the traditional methods of data collection and analysis using surveys and other means cannot capture such timely and large-scale data; these methods also have other disadvantages.^18^ Therefore, some studies draw on big data. For example, Wong et al^19^ analysed relative search volume data on the topic “surgical masks” by Google Trends. Alomari et al^18^ studied government pandemic measures and netizen tweets posted on Twitter. At present, articles addressing government policies and measures on public behaviour by analysing big data are limited, and existing studies mainly focus on single behaviour. Weible et al^20^ analysed COVID-19 and the related policy sciences. They then concluded that more research is needed to examine the relationship between crises and public responses, given the necessity for mass behavioural change to overcome the pandemic.

 On the one hand, this study assesses the effects of the governments’ epidemic measures on public behaviour. This is achieved by combining big data, which is open to the public by multiple authorities, and visualising the hybrid data, in order to reflect behaviour changes. Specifically, three types of data that indicate key behaviour changes in the public (namely levels of concern regarding the epidemic, self-protection behaviour, and mobility trends) are aggregated. On the other hand, this study also compares three different countries, namely China, Italy, and the United States, which are the countries that had the most cases in Asia, Europe, and North America, respectively, within the first three months after the first case was reported. Considering that population movements between countries were severely restricted, the scope of mobility trends was narrowed to the three specific areas that had the largest number of confirmed cases in the three countries during the same period mentioned above. Specifically, those three areas were Hubei Province of China, Lombardy of Italy, and New York State of the United States.

## Methods

###  Hybrid Large-Scale Data Source Related to Public Behaviour

 This paper summarizes large-scale data related to changes in public behaviour during the epidemic in China, Italy, and the United States. The data were taken from different databases provided by multiple authorities, all of which are open to the public. These sources are Baidu Migration, Baidu Search, Alibaba Index, Google Search, Google Mobility Trend, and Apple Mobility Trend. The data include keyword searches, purchasing indexes, shopping searches related to purchase records and population mobility during the epidemic. Three types of these data, as shown in the category of data in Table, reflecting behavioural changes were selected as indexes of epidemic concern, self-protection, and mobility trends. The data of epidemic concern and self-protection cover the whole area of the three countries, whereas the data of human mobility trends only indicate the specific infected region mentioned in each country.

**Table:** Basis of Large-Scale Data Collection on Public Behaviour

**Public Behaviour**	**Data Platform**	**Data Category**	**Search Terms**
Epidemic concern	Google (US, Italy)	Search interest	COVID-19, coronavirus
	Baidu (China)		新冠, 不明原因肺炎
Self-protection	Google (US, Italy)	Shopping search interest	Face Mask, hand sanitizer
	Google (Italy)	Shopping search interest	Mascherin, igienizzante mani
	Alibaba (China)	Purchasing index	口罩, 洗手液
Mobility trends	Google (US, Italy)	Global mobility report	Public transport hubs
	Baidu (China)	Baidu mobility index	迁入, 迁出

Abbreviation:COVID-19, coronavirus disease 2019 *Notes:*(1) Before January 9, 2020, in China, “unexplained pneumonia” (不明原因肺炎) was used as a substitute for “coronavirus” (新冠) in search terms, since the virus had not yet been identified. In addition, COVID-19 was not confirmed by the WHO before February 11, 2020. Therefore, coronavirus was also used as the search term in this study. (2) China’s self-protection is analysed through the data of actual purchase behaviour provided by Alibaba, while the self-protection of the United States and Italy is analysed by adopting Google’s shopping search index instead of actual shopping behaviour. This is due to not having found other appropriate platforms for obtaining open shopping data for the latter two countries. (3) In order to be consistent with Google’s data format, the specific values provided by Baidu Search and Ali Index are converted to percentages. Specifically, in order to eliminate the data errors caused by China’s lunar new year’s massive population flow, the Baidu Migration daily value is converted into a percentage, compared with the baseline value taken from the same period in the previous year, based on the lunar calendar.

###  Dataset of Governmental Health Responses and Mobility Restrictions

 Since the outbreak of the COVID-19 epidemic, different countries have released various health measures on their official websites or authoritative health agency websites. This study has manually collected 490 items of epidemic prevention and control information released by governmental and authoritative health agency websites, of which 128 were from China (in Chinese, December 31, 2019-March 15, 2020), 107 from Italy (in Italian and English, January 23, 2020-May 3, 2020), and 255 from the United States (in English, January 8, 2020-May 3, 2020). Detailed information about the governmental responses and website sources of each country are listed in Table S1 (see Supplementary file 1). All this information is divided into three categories: epidemic situation (eg, updates on cases, areas), health guidelines (eg, health advice to people and agencies) and policy measures (eg, mobility restrictions, testing work plans, and financial and resources supply plans). The time span of information collection covers from the time the epidemic concern index began to change significantly for the first time, to the end of the first round of regional lockdowns. The policy information collected from the above channels is used as the dataset of governments’ health responses throughout the whole of this research.

 Among all policy measures, mobility restrictions are of key importance. The mobility restrictions of the three abovementioned countries are studied through a content analysis method and qualitative coding. The codebook of mobility restrictions is shown in Table S2A. A comprehensive evaluation of the restrictions’ severity was then carried out in eight dimensions, namely regional lockdown, local public transport closure, residential area control, public places closure, outdoor activities ban, penalties for violating the ban, army enforcement, and information technology-based control measures. The scoring criteria for mobility restrictions are shown in Table S2B. Each of the two indicators (the implementation time and the intensity of a specific measure in terms of restricting mobility) accounts for half the weight of the scores. Taking into consideration the significant regional differences in mobility restrictions and public mobility behaviour in these three countries, the three regions with the most cases within the first three months after the first case was reported were selected for comparative study: Hubei province of China, the Lombardy region of Italy, and New York state of the United States. Since these three regions have maintained similar leading positions in terms of both economy and population in their respective countries, they are representative and comparable. Forty policies were randomly selected for an inter-rater reliability test, and the results showed high consistency between the two raters.

###  Hybrid Data Processing and Visualization

 All the original data were captured by Python from the open data platforms listed in Table. Then, the data were analysed by Excel to draw graphs by Origin and Inkscape. In order to facilitate the comparison of data from different regions and platforms, the values are the relative ratios with their previous changes.

**Figure 1:**
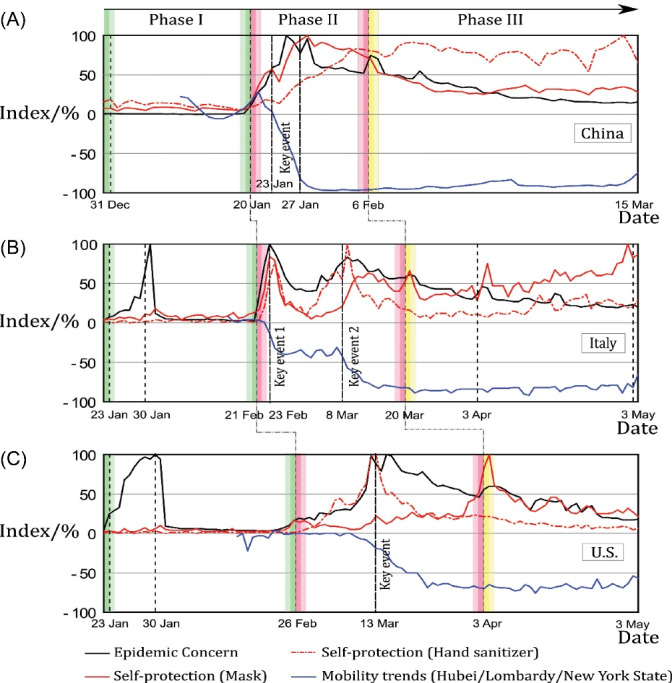
Time-Index Graph of the Effect of Government Epidemic Management Events. *Notes:*(1) Indexes of epidemic concern and self-protection show the daily values for search popularity. These values have been converted into percentages relative to the highest value within a specific period. The index of mobility trends shows the percentage change between the daily value and the baseline value. (2) The term ‘key event’ refers to a typical event where all three indicators (epidemic concern, self-protection behaviours, and mobility trends) reached their highest peak. China (**Panel A**): The lockdown measure in different cities in Hubei province from January 23, 2020 to January 27, 2020 is regarded, as a whole, to be China’s key event. Italy (**Panel B**): Two lockdown measures with different ranges on the 23^rd^ of February and the 8^th^ of March caused simultaneous peaks in the epidemic concern and self-protection index on those very days. These peaks were coupled with two shocks in the mobility trends. US (**Panel C**): The key event is the declaration of a state of emergency by the President of the United States of America on March 13, 2020. (3) The population mobility data were available on the above platforms after the outbreak of the epidemic. Therefore, the population mobility data (blue line) failed to start from the starting point.

 When processing hybrid source data to reflect behavioural changes, the large-scale data from different platforms were selected as indexes of epidemic concern, self-protection, and mobility trends. In particular, indexes of epidemic concern and self-protection present the ratio of the absolute value of daily search popularity to the highest value in the selected time period. The index of mobility trends presents the relative ratio with the baseline value of normal days without COVID-19 in the given countries. All the indexes reflecting behavioural changes are integrated into a time-index graph. After adding important corresponding health measure events by date into this graph, the significant impact of health measures on public behaviour changes can be clearly seen. The time-index graph of the effect of government measures on public behaviour is shown as Figure 1, and the data analysis of the behaviour changes caused by specific health measures is listed in Table S3.

 To further analyse the relation between the mobility trends and the strictness of governmental measures, the above method of data processing has been extended. Specifically, the average value of the daily decreasing mobility trend was used, with a total selection of 30 days of data after the blockade in each region. This study also conducted a Pearson correlation test on these two variables, the mobility trends and the strictness of governmental measures, by using SPSS 26.

## Results

 As a time-index graph, Figure 1 shows the changes in public behaviour around the outbreak of the epidemic and the corresponding relationship with policy measures. The horizontal lines of epidemic concern, self-protection, and mobility trends changed dramatically and formed three distinct stages. Events that caused significant changes are marked in dotted vertical lines. The specific behaviour changes and the corresponding policy items are shown in Table S3.

###  Three-Phase Rule of Public Behaviour in an Epidemic

 Governmental health measures had profound impacts on public behaviour. As can be seen from Figure 1, however, during the COVID-19 epidemic, the governmental measures did not immediately persuade the public to change their behaviours. Instead, the public’s behaviour proceeded in a three-phase process, namely the wait-and-see phase (Phase I), the surge phase (Phase II), and the slow-release phase (Phase III). In one day, the black line (showing the rate of concern about the epidemic) increased by more than 100% from the previous day. This was taken as the start of the wait-and-see phase. Similarly, when the absolute value of the index of epidemic concern, self-protection and mobility trends (black line, red line and blue line, respectively) all increased by more than 100% from the previous day, this was taken as the start of the surge phase. When the change degree of epidemic concern, or the self-protection index, mentioned increased again by more than 100% from the previous day, while the mobility trends remained steady, that date was taken as the start of the slow-release phase.

###  Phase I: Wait and See Phase (Dominated by Consciousness Change Only)

 Early events, such as the first reported case and subsequent escalating alerts, caused people to pay close attention to the epidemic. However, self-protection behaviour could not be immediately aroused. The epidemic concern (black line) quickly reached its first peak when it reached 100% in Italy (a +54% increase over the previous day) on January 31, 2020, and 100% in the United States (+6% increase) on January 30, 2020. As the first case was reported by China, the spread of the virus was considered to be an ordinary commotion and thus did not attract much attention in the beginning. The early result was only a 0.39% epidemic concern increment in China on December 31, 2019. In contrast, there was no one concerned about the epidemic on the previous day.

###  Phase II: Surge Phase (Dominated by Both Consciousness and Behaviour Changes)

 Phase II was marked by significant changes in public behaviour. These changes were caused by events such as the upgrading of epidemic warnings and the implementation of mobility restrictions, as listed in Table S3. Among all government measures, regional blockades and declaration of emergency sent the strongest epidemic signal that led to the most drastic changes in local public behaviour. Therefore, these events are regarded as key events; such events caused most of these indices to reach their highest peaks. The epidemic concern index reached 100% (+59% increase) in China on January 25, 2020; 100% (+37%) in Italy on February 23, 2020, reached 100% (+27%) in the United States on March 15, 2020. The mask wearing self-protection index (red line) reached 100% (+6%) in China on January 28, 2020. In Italy, the self-protection index (wearing masks) reached 84% (+105%) on February 23, 2020, and the self-protection index of using hand sanitizer (dotted red line) reached 100% (+82%) on March 9, 2020. In the United States, the self-protection index (hand sanitizer) reached 100% (+4%) on March 13, 2020. As to the mobility trend, it decreased in Hubei by about 90% on January 28, 2020; by 30% on February 24, 2020, and by 80% on March 14, 2020 in Lombardy. In New York State, the mobility trend decreased by 60% on March 23, 2020. When the index of epidemic concern and self-protection reached their peaks, a half-month period of decline generally followed when no external causes were identified. However, even after falling back the indices were still higher than they had been before the key events.

###  Phase III: Slow-Release Phase (When New Balance Is Established in Both Consciousness and Behaviour )

 The dramatic changes brought about by the epidemic showed a moderate trend at this stage. New events, such as the release of clear, targeted epidemic prevention guidelines and of reopening policies, raised the epidemic concern and self-protection indices once more, whereas the mobility trends index remained steady at a low level. The specific events varied from country to country but included recommended requirements (eg, health guidelines), mandatory requirements (eg, policy measures), and spontaneous requirements (eg, reopening decisions). In Italy, when the policy entitled “The Urgent Measures Regarding the Containment and Management of the Epidemiological COVID-19 Emergency” expired on May 3, 2020, people returned to work. As a type of spontaneous requirement, this expiration instead caused the self-protection index (mask) to increase to the highest value of 100%.

###  Effects of Mobility Restrictions

**Figure 2:**
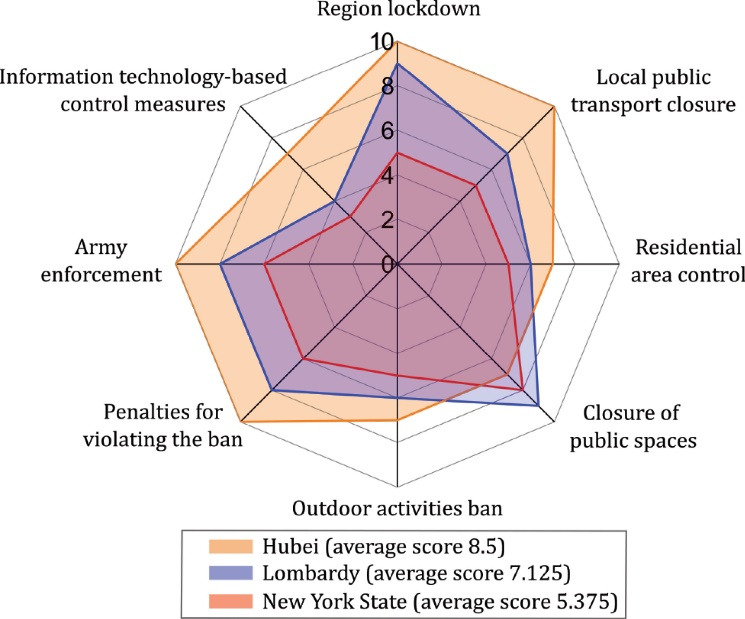
Radar Score Chart of Mobility Restriction Measures. **Note: **Key Pronouncements on mobility restrictions include the “Government Epidemic Prevention and Control Command Series Notice (January 23-27)” in Hubei Province, China, “Take urgent measures regarding the containment and management of the epidemiological COVID-19 emergency decree (February 23 Feb and March 8)” in Lombardy, Italy, and “New York State on PAUSE Executive Order (March 20)” in New York state, USA.

 Based on the specific scores for the individual items for each country in Table S2C, the data for the strictness of mobility restriction in each country are plotted into Figure 2. The three countries can be ranked according to the degree of strictness as follows: Hubei province in China (with an average score of 8.5 out of 10), Lombardy in Italy (7.125), and New York state in the United States (5.375).

**Figure 3:**
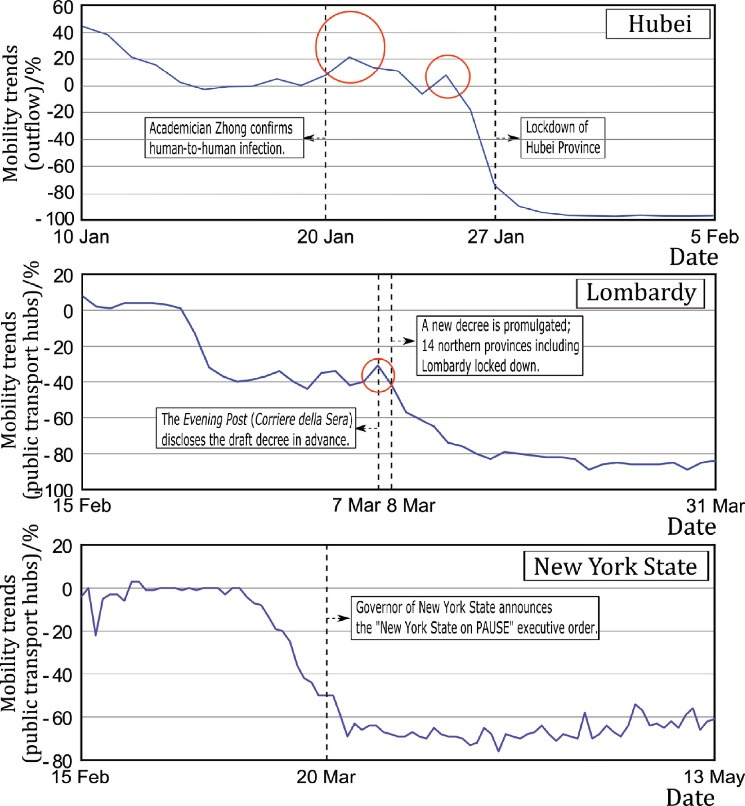
Comparison of the Effect of Mobility Restrictions.

 The varying stringency of mobility restrictions (Figure 2) led to differences in the restrictions’ short-term effects and long-term effects. The government releasing key epidemic information can cause an instantaneous outflow of population from the epidemic area (Figure 3). In China, when Academician Nanshan Zhong confirmed on January 20, 2020, that human-to-human transmission of the coronavirus was possible, an immediate wave of population outflow (here referred to as a “surge”) followed closely. This led to the arrival of Phase II in Hubei, China (Figure 3). This finding indicates that the release of information describing the seriousness of the epidemic can lead to a surge. For example, in the city of Wuhan, the first city to be locked down in Hubei province, the first lockdown announcement was made on January 23, 2020. Subsequently, nearly all cities in Hubei Province issued lockdown measures during the time period from January 23-27, 2020. During this time, the outflow of population from Hubei Province increased again, forming a significant surge, as shown in the red circle in Figure 3. In Italy, urgent measures regarding the containment of COVID-19 were released on March 8, 2020. These mobility restrictions were more detailed, broader, and stricter than the measures previously released on February 23, 2020. One day before the official implementation of the March 8th decree, the Evening Post (Corriere della Sera) of Milan disclosed the draft decree in advance, causing panic in Lombardy. There was also an obvious surge in the population outflow through public transport hubs in Lombardy on March 7, 2020.

**Figure 4:**
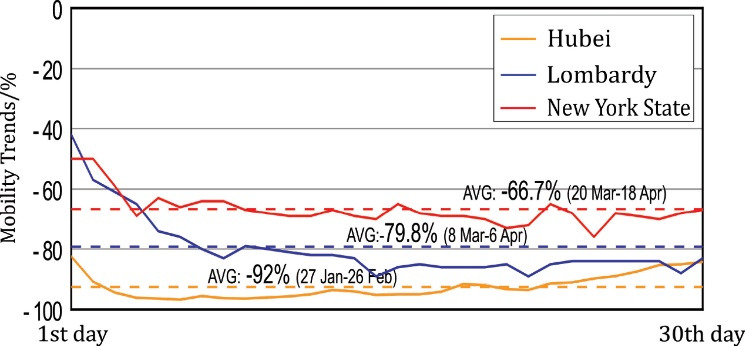
Comparison of the Average Value of the Decreasing Mobility Trend Within 30 Days After the Implementation of Mobility Restrictions.

 Since the shortest time in the three regions from the implementation of mobility restriction measures to the decision to reopen was about one month, this study calculates the average value of the mobility trend change within 30 days after the mobility restrictions were implemented, as shown in Figure 4.

 In China’s Hubei province, which had the strictest mobility restrictions among the three countries, the average reduction in population mobility within 30 days was 92%. This was followed by Lombardy, Italy, with an average reduction of 79.8%. Finally, New York state of the United States, with the most relaxed mobility restrictions, had an average mobility reduction of 66.7%. The Pearson correlation test in Table S4 shows that the long-term effect caused by mobility restriction measures is significantly positively related to their degree of rigor, with a correlation coefficient of 0.88 at the level below 0.01.

###  Effect of Self-protection Guidelines

 With regard to self-protection guidelines, the three countries differ in their epidemic experiences, population, social culture, and living habits. The timing and content of recommendations were different in each case, leading to different effects on changes in the public’s self-protection behaviour. The following analysis takes mask-wearing as an example.

 Because of prior experience with the SARS epidemic, China attaches great importance to masks as a form of protection against this type of respiratory disease. Therefore, wearing masks in public was made mandatory in most areas in China. As a result, the self-protection index (mask) showed an overall increase from the beginning of Phase II, reaching 100% on January 28, 2020 (Figure 1 and Table S3A).

 Due to the long latency and highly infectious nature of the coronavirus, the US Centers for Disease Control and Prevention (CDC) issued a mask guideline on April 3, 2020, encouraging people to make their clothes into face masks, in order to avoid crowding out medical resources. This occurred during the slow-release phase. At that time, even though experts doubted the efficacy of wearing home-made masks, the prevalence of mask wearing abroad and news about the rapid spread of the epidemic had a strong and immediate effect. The self-protection index (mask) quickly reached 100% on April 4, 2020 (Figure 1 and Table S3C).

 Italy has not placed much emphasis on mask wearing as a prevention measure. Like the United States, Italy has emphasized social distancing as one of its major protection guidelines. Therefore, the self-prevention index (mask) in Italy did not reach its 100% peak until Italy began to reopen regions at the end of Phase III on May 3, 2020. The self-protection index (mask) in Italy reached 100% on May 2, 2020, largely due to the public’s spontaneous need to return to work and to go outdoors (Figure 1 and Table S3B).

## Discussion

 This research explores the effect of governmental health measures on public behaviour during the outbreak of the COVID-19 epidemic in three countries from different continents that experienced severe early outbreaks. The outcomes of health measures and public behaviour evolved in a continuous process, with common characteristics.

 First, this study reveals that, during the COVID-19 epidemic, the effect of government measures on public behaviour played out following a three-stage rule. In the wait-and-see phase, the first report of the epidemic and the escalation of alerts usually caused people to pay close attention to the epidemic situation. However, their behaviour did not change immediately. As described by the stages-of-change theory,^21,22^ behaviour change is a process, rather than a single event. Behaviour usually proceeds through several steps in order to achieve lasting change. Therefore, significant changes to public behaviour in the surge phase and the slow-release phase occurred in response to certain events, such as the upgrading of epidemic warnings, the implementation of mobility restrictions, regional blockades, the release of epidemic prevention guidelines and reopening policies. In addition, Uddin et al^23^ found that the implementation of measures was mostly inﬂuenced by the infection rate. Moreover, as Brzezinski et al^24^ discovered, individuals engaged in physical distancing (even in the absence of lockdown policies), once the virus took hold in their area. Therefore, the increased severity and infection in the latter two phases may also have contributed to policy-making, as well as the corresponding behavioural changes. However, isolation and protective procedures were less effective as a tidal wave of cases accrued. Therefore, the optimization of the treatment plan and the development of specific drugs would be of greater importance.^6^

 Second, this research uncovers and analyses the surge phenomenon after regional blockades in the short term, as well as the relationship between mobility behaviour and policies in the long-term. When either the epidemic is described as serious or when mobility restrictions are stringent, the outflow of population from the epidemic area will be accelerated in a short time, forming a surge. The stricter the policy is, the more serious the surge phenomenon of population outflow from the epidemic area in the short term will be. As for the long term, although the international pandemic situation and the measures of other countries were open to the world, the effect of mobility restrictions was positively related to the stringency of local policies. This indicates that, even in an information age, public behaviour is still deeply affected by the actions of local governments, rather than the global situation. This study found that much of the decline in the epidemic curve was driven by critical behaviour changes within local communities, rather than by international efforts.^10^ Besides, Muto et al^16^ explored what kinds of information affected public behavioural changes. The study discovered that the local government is the most trusted source of information. Among all local governmental measures, a regional blockade was the strongest epidemic signal that led to the most drastic changes in local public behaviour in all indices. One survey suggests that attitudes vary in line with the nature of interventions, ie, the provision of information is more acceptable to the public than regulations to limit their behaviours.^25^ As these epidemic restrictions deeply interfere with everyone’s lives, it is possible that severe restrictions could lead to a popular non-acceptance and revolt, resulting in the surge phenomenon. In addition, mass quarantining is considered to be one of the most effective methods for controlling the spread of COVID-19.^26,27^ However, as shown in this study, a mild mobility ban will not definitely lead to a surge, but weak bans are not as effective as strict bans in achieving a long-term reduction in mobility. Moreover, an analytic formula for the efficiency of intervention strategies shows that the efficiency of an intervention strategy decays quite quickly as the adoption time is delayed.^28^ Therefore, the release and implementation of a mobility ban should be as immediate as possible.

 Third, contextual differences, such as epidemic experience, social culture, and lifestyle habits may affect epidemic management and the effects of that management in different countries. Landoni et al^29^ analysed why Asian countries are outperforming the Western world. The study stated that previous experience of epidemics in the modern era, social acceptance of physical distancing and face masks are influencing factors. Olagnier and Mogensen^30^ emphasized the importance of the contribution of social heritage and culture to the effective management of the coronavirus crisis in Denmark. Specifically, they said that Danish people have few physical contacts, and Danes like to keep a decent social distance compared to Italians, French and Spanish. It is worth noting that, in addition to the differences between countries, socio-demographic status and personal attributes can also influence compliance with COVID-19 preventive behaviours.^31^ For example, mask use differs significantly by age group and gender.^15^ Future research could explore the influencing factors more comprehensively; future policies also need to be more targeted. Furthermore, no matter when and how countries begin to require citizens to wear masks, and whether people in various countries increase their mask-wearing behaviour due to mandatory requirements (eg, China), recommended requirements (eg, the United States), or spontaneous requirements (eg, Italy), the public will eventually show a strong change in their online searches related to mask purchases (Figure 1). This finding indicates that, under the influence of the real-time and globalized exchange of information, the final effects will still converge.

 Overall, based on hybrid large-scale data, this paper tests the effects of policy through an analysis of public behavioural change. Lazarus et al^32^ have developed the COVID-19 assessment scorecard (COVID-SCORE), which is a list of 19 statements that enable anyone to conduct an easy assessment of their government’s response to COVID-19. Using the objective behavioural data in this paper, a similar scorecard could also be formed in the future to assess the policies of different countries. Furthermore, Alam et al^33^ proposed a technology-driven framework, named iResponse, for coordinated and autonomous pandemic management. The iResponse framework allows for pandemic-related monitoring and policy enforcement, resource planning and provision-making, and data-driven planning and decision-making. In general, the use of new technologies, such as AI and big data, is a trend in epidemiological management.

###  Limitations

 Based on both a combination and visualization of hybrid data sources, this study makes use of big data collected and reported by multiple authorities. Regrettably, this type of research method has its own limitations, such as data noises, and the authenticity, timeliness, completeness, representativeness and legality of the data. The same type of data comes from multiple platforms with different standards. However, some transformations have been made to unify the data types, such as from absolute value to relative value, where the differences in rules between data fetches and platforms may cause some differences in results. One example of data noises is that Google’s mobility trend data showed that the population mobility of New York State was greatly reduced on Presidents’ Day in the United States (February 17, 2020), which was a public holiday. Such factors could be mistakenly attributed to the effects of the government’s health measures.

 When carrying out this study, we tried our best to find convincing data platforms, but this does not mean that the platforms used provide the best data required for this study. Further, we cannot rule out that other platforms can provide more appropriate public data. Furthermore, the data adopted in this research are all incomplete sets of public data from mainstream platforms, and they only cover a limited time span. In future research, more cooperation with government agencies could be a way to develop more targeted data resources and thus obtain more precise results. In addition, operational errors could possibly occur in manual qualitative coding of health measures.

## Conclusion

 From this study, one can find that public behaviour is deeply affected by the actions of their local governments, rather than the global epidemic situation. The governmental health measures did not immediately persuade the public to change their behaviours during the COVID-19 epidemic outbreak. Rather, the public behaviour proceeded the three-phase rule that typically occurs in an epidemic outbreak. Strict mobility restrictions are more likely to cause a surge of population outflow from the epidemic area in the short term, whereas the effect of mobility restrictions is positively related to the stringency of policies in the long term.

## Ethical issues

 Ethical approval for this type of study is not required by our institute.

## Competing interests

 Authors declare that they have no competing interests.

## Authors’ contributions

 Conception and design (GYW, LL); Data collection and analization (LL); Drafting of the manuscript (All authors); Critical revision of the manuscript (GYW, LL, and LFW).

## Funding

 The study is supported by the project of National Social Science Youth Fund of China (grants 21CSH074).

## Supplementary files

Supplementary file 1 contains Tables S1-S4.
