# Supplementary material for: The Effect of Governmental Health Measures on Public Behaviour During the COVID-19 Pandemic Outbreak
Source: Int J Health Policy Manag. 2021 Sep 11;11(10):2166–74. doi: 10.34172/ijhpm.2021.131 (PMC9808271; doi:10.34172/ijhpm.2021.131)
Supplement: Supplementary file 1 — contains Tables S1-S4. [file ijhpm-11-2166-s001.pdf]

Article title: The Effect of Governmental Health Measures on Public Behaviour During the COVID-19 Pandemic Outbreak

Journal name: International Journal of Health Policy and Management (IJHPM)

Authors' information: Guoyan Wang[1]*, Li Li[2]*, Lingfei Wang[1], Zhi Xu[3]

[1]School of Communication, Soochow University, Suzhou, China.

[2]Health Inspection Institute, Health Commission of Suzhou, Suzhou, China.

[3]Jiangsu Key Laboratory of Culture and Tourism for Digital Twin Perception Technology in Museums, Suzhou, China.

(*Corresponding authors: Guoyan Wang; Email: gywang@suda.edu.cn & Li Li; Email: lilysz0828@163.com)

Supplementary file 1

Table S1. Information collection of governments' health responses to COVID-19

**Items/Country**	**China**	**Italy**	**United States**	**Total**
Language	Chinese	Italian & English	English	
Time span	Dec 31, 2019 - Mar 15, 2020	Jan 23, 2020 - May 3, 2020	Jan 8, 2020 - May 3, 2020	
Number of epidemic situation reports	68	46	121	235
Number of heath guidelines	28	16	109	153
Number of policy measures (mobility restrictions)	32 (20)	45 (27)	25 (13)	102 (59)
Total number of information	128	107	255	490

Notes:
(1) Sources of Chinese data:
Chinese Centre for Disease Control and Prevention (http://www.chinacdc.cn/)
National Health Commission of China (http://wjw.hubei.gov.cn/bmdt/ztzl/fkxxgzbdgrfyyq/)
Health Commission of Hubei Province (http://www.nhc.gov.cn/xcs/xxgzbd/gzbd_index.shtml)
Wuhan Municipal Health Commission (http://wjw.wuhan.gov.cn/ztzl_28/fk/yqtb/).
(2) Sources of Italian data:
Italian Government Presidency of the Council of Ministers (http://www.governo.it/it/coronavirus-misure-del-governo)
Italian Ministry of Health (http://www.salute.gov.it/portale/nuovocoronavirus/homeNuovoCoronavirus.jsp?lingua=english)
Epidemiology news for public health in Italian (https://www.epicentro.iss.it/en/coronavirus/news)
(3) Sources of United States data:
U.S. Government Response to COVID-19 (https://www.usa.gov/coronavirus)
U.S. Department of Health & Human Services (https://www.hhs.gov/)
U.S. Centers for Disease Control and Prevention (https://www.cdc.gov/coronavirus/2019-ncov/whats-new-all.html)
New York State Government (https://www.ny.gov/)

Table S2. Coding and scoring of mobility restrictions

Table S2A. Codebook of mobility restrictions

Encoding name	Detailed description	Inclusion criteria	Exclusion criteria	Typical exemplars	Atypical exemplars	"Close, but no"
Lockdown	Regional lockdown	Time and intensity of implementation	/	Start time of the restriction; mandatory or recommended; whether the code is involved in the specific measures	"Stay at home"	"Maintain social distance"
Closure	Local public transport closure					
	Public places closure					
Ban	Outdoor activities ban					
Control	Residential area control					
	Information technology-based control measures				/	/
Penalties	Penalties for violating the ban					
Enforcement	Army enforcement					

Note: According to the sample codebook template by Saldaña,
Saldaña, Johnny, 2016. The Coding Manual for Qualitative Research (3rd edition). Thousand Oaks, CA: SAGE Publications (page 28).

Table S2B. Scoring criteria for mobility restrictions

**Implementation time (5 points)**		**Implementation intensity (5 points)**	
Criteria	Score	Criteria	Score
Earlier than key event or key event itself	5	Mandatory	5
Within 3 days after key event	4	Strongly recommended	4
3-15 days after key event	3	Recommended	3
15 days to 1 month after key event	2	Involved and partially recommended	2
1 month - 2 months after key event	1	Involved but not recommended	1
More than 2 months after key event	0	Not involved	0

Table S2C. Mobility restriction scores of three countries in eight dimensions (10-point system)

**Scoring dimension**	**Hubei score**	**Lombardy score**	**New York State score**
Regional lockdown	10<br>(23-27 Jan, mandatory)	9<br>(23 Feb & 8 Mar, mandatory)	5<br>(Stay-at-home recommendation)
Local public transport closure	10<br>(25 Jan, mandatory)	7<br>(22 Mar, mandatory)	5<br>(Stay-at-home recommendation)
Residential area control	7<br>(14 Feb, mandatory)	6<br>(23 Feb & 8 Mar, mandatory)	5<br>(Stay-at-home recommendation)
Public places closure	7<br>(16 Feb, mandatory)	9<br>(23 Feb & 8 Mar, mandatory)	8<br>(20 Mar, mandatory)
Outdoor activities ban	7<br>(16 Feb, mandatory)	6<br>(20 Mar, mandatory)	5<br>(Stay-at-home recommendation)
Penalties for violating the ban	10<br>(Investigate criminal responsibility according to the law; intentional spreaders receive the highest sentence of death)	8<br>(Violators of the closure order can be charged, with a maximum fine of 3000 euro)	6<br>(Violations of the home order are punishable by a fine of 250-500 USD)
Army enforcement	10<br>(23 Jan, enforcement)	8<br>(9 Mar, enforcement)	6<br>(13 Mar, general epidemic prevention and treatment)
Information technology-based control measures	7<br>(22 Feb, mandatory)	4<br>(16 Apr, recommended)	3<br>(16 May, recommended)
Total score	68	57	43
Average score	8.5	7.125	5.375

Table S3. Effects of government health measures in three countries

Table S3A. Effects of government health measures in China

<table>
<tr><td rowspan="2">Category of data</td><td colspan="4">Phase I</td></tr>
<tr><td>Start I (Date-event)</td><td>Peak I (Date-event)</td><td>Peak I (Date-index & rate of change)</td><td>Reduce (R) or stable (S) /reduction period (days)</td></tr>
<tr><td>Epidemic concern</td><td rowspan="4">31 Dec 2019: Wuhan Health Commission for the first time reports unexplained viral pneumonia.</td><td rowspan="4">31 Dec 2019: Wuhan Health Commission for the first time reports unexplained viral pneumonia.</td><td>31 Dec 2019
0.39%
(Previous day was 0)</td><td>R/1</td></tr>
<tr><td>Self-protection (Mask)</td><td colspan="2">NA</td></tr>
<tr><td>Self-protection (Hand sanitizer)</td><td colspan="2">NA</td></tr>
<tr><td>Mobility trends of Hubei Province</td><td colspan="2">NA</td></tr>
<tr><td rowspan="2">Category of data</td><td colspan="4">Phase II</td></tr>
<tr><td>Start II (Date-event)</td><td>Key event (Date-event)</td><td>Peak II (Date-index & rate of change)</td><td>Reduce(R) or stable(S) / reduction period (days)</td></tr>
<tr><td>Epidemic concern</td><td rowspan="4">20 Jan 2020: Academician Nanshan Zhong confirms human-to-human transmission of the coronavirus for the first time.</td><td rowspan="4">23-27 Jan 2020: Lock down of all cities in Hubei Province.</td><td>25 Jan
100%
(+59%)</td><td>R/11</td></tr>
<tr><td>Self-protection (Mask)</td><td>28 Jan
100%
(+6%)</td><td>R/21</td></tr>
<tr><td>Self-protection (Hand sanitizer)</td><td colspan="2">Steadily increasing</td></tr>
<tr><td>Mobility trends of Hubei Province</td><td>28 Jan+
-90%↑
(-11 %)</td><td>S/-</td></tr>
<tr><td rowspan="2">Category of data</td><td colspan="4">Phase III</td></tr>
<tr><td>New event 1 (Date-event)</td><td>Peak III 1 (Date-index)</td><td>New event 2 (Date-event)</td><td>Peak III 2 (Date-index & rate of change)</td></tr>
<tr><td>Epidemic concern</td><td rowspan="4">6 Feb 2020: A doctor in Wuhan dies of coronavirus.</td><td>6 Feb
75%
(+44%)</td><td rowspan="4">13 Feb 2020: Hubei Province reports 13,332 clinical cases for the first time.</td><td>13 Feb
55%
(+22%)</td></tr>
<tr><td>Self-protection (Mask)</td><td>NA</td><td>NA</td></tr>
<tr><td>Self-protection (Hand sanitizer)</td><td>NA</td><td>12 Feb
91%
(+1%)</td></tr>
<tr><td>Mobility trends of Hubei Province</td><td>NA</td><td>NA</td></tr>
</table>

Table S3B. Effects of government health measures in Italy

**Category of data**	**Phase I** Start I (Date-event)	Peak I (Date-event)	PeakI(Date-index & rate of change)	Reduce (R) or Stable (S) /reduction period (Days)
Epidemic concern	23 Jan 2020: State of emergency announced.	30 Jan 2020: WHO declares an international emergency.	31 Jan 100% (+54%)	R/1
Self-protection (Mask)			1 Feb 18% (+80%)	R/2
Self-protection (Hand sanitizer)			1 Feb 4% (+100%)	R/2
Mobility trends of Lombardy			NA	

**Category of data**	**Phase II** Start II (Date-event)	Key event 1 (Date-event)	Peak II 1 (Date-index & rate of change)	Reduce (R) or stable (S)/reduction period (Days)	Key event 2 (Date-event)	Peak II 2 (Date-index & rate of change)	Reduce (R) or stable (S) /reduction period (Days)
Epidemic concern	21 Feb 2020: The Ministry of Health issues the decree on compulsory quarantine.	23 Feb 2020: 11 towns are locked down in Lombardy and Veneto.	23 Feb 100% (+37%)	R/9	8 Mar 2020: A new decree to contain and manage the emergency situation is announced, and 14 northern provinces, including Lombardy, are locked down.	9 Mar 83% (+8%)	R/11
Self-protection (Mask)			23 Feb 84% (+105%)	R/8		13 Mar 63% (+11%)	R/6
Self-protection (Hand sanitizer)			24 Feb 77% (+40%)	R/6		9 Mar 100% (+82%)	R/6
Mobility trends of Lombardy			24 Feb -30%↑ (-146%)	S/-		14 Mar -80%↑ (-5%)	S/-

**Category of data**	**Phase III** New event 1	Peak III 1 (Date-index)	New event 2 (Date-event)	Peak III 2 (Date-index &	New event 3 (Date-event)	Peak III 3

	(Date-event)			rate of change)		(Date-index& rate of change)
Epidemic concern	20 Mar 2020: The highest number of deaths in a single day.<br>21 Mar: The largest increase in newly-diagnosed cases in a single day.	21 Mar 61% (+7%)	3 Apr 2020: U.S. CDC releases guidance for cloth face coverings.	4 Apr 46% (+28%)	3 May 2020: The decree to contain and manage the COVID-19 emergency expires.	NA
Self-protection (Mask)		21 Mar 66% (+29%)		5 Apr 75% (+53%)		2 May 100% (+30%)
Self-protection (Hand sanitizer)		NA		NA		NA
Mobility trends of Lombardy		NA		NA		4 May -60%↑ (+21%)

Table S3C. Effects of government health measures in the United States

Category of data	Phase I			
	StartI (Date-event)	Peak I (Date-event)	Peak I (Date-index & rate of change)	Reduce (R) or stable (S) /reduction period (Days)
Epidemic concern	21 Jan 2020: The first case confirmed in the country.	30 Jan 2020: The CDC confirms the first human-to-human transmission. 31 Jan 2020: The HHS declares a public health emergency.	30 Jan 100% (+6%)	R/2
Self-protection (Mask)			31 Jan 10% (+67%)	R/1
Self-protection (Hand Sanitizer)			30 Jan 3% (+200%)	R/1
Mobility trends of New York State			NA	
**Category of data**	**Phase II**			
	Start II (Date-event)	Key event (Date-event)	Peak II (Date-index & rate of change)	Reduce (R) or stable (S) /reduction period (Days)
Epidemic concern	26 Feb 2020: The CDC reports the first case of community transmission.	13 Feb 2020: Declaration of state of emergency by POTUS	15 Mar 100% (+27%)	R/18
Self-protection (Mask)			13 Mar 22% (+69%)	R/3
Self-protection (Hand Sanitizer)			13 Mar 100% (+3%)	R/9
Mobility trends of New York State			23 Mar -60% (-17%)	S/-
**Category of data**	**Phase III**			
	New event 1 (Date-event)	Peak III 1 (Date-Index)	New event 2 (Date-event)	Peak III 2 (Date-index & rate of change)
Epidemic concern	3 Apr 2020: The CDC releases guidance for cloth face coverings.	4 Apr 60% (+7%)	16 Apr 2020: The President releases "Opening Up America Again" guidelines.	NA
Self-protection (Mask)		4 Apr 100% (+20%)		16 Apr 46% (+28%)
Self-protection (Hand Sanitizer)		NA		NA
Mobility trends of New York State		NA		2 May -50%↑ (+16%)

Table S4. Pearson correlation test

Correlation	Items	Mobility restriction	Decreasing mobility
Mobility restriction	Correlation coefficient	1	.880**
	Sig.		.000
	Cases	90	90
Decreasing mobility	Correlation coefficient	.880**	1
	Sig.	.000	
	Cases	90	90

The two-tailed test shows a significant correlation at the level below 0.01.
